# Waterborne Polyurethane Coatings with Covalently Linked Black Dye Sudan Black B

**DOI:** 10.3390/ma10111247

**Published:** 2017-10-28

**Authors:** Tao Wang, Wei Sun, Xingyuan Zhang, Haiyan Xu, Fei Xu

**Affiliations:** CAS Key Laboratory of Soft Matter Chemistry, Department of Polymer Science and Engineering, University of Science and Technology of China, Hefei 230026, China; wt0510@mail.ustc.edu.cn (T.W.); kdsw@mail.ustc.edu.cn (W.S.); sa12226023@mail.ustc.edu.cn (H.X.); puglobe@mail.ustc.edu.cn (F.X.)

**Keywords:** Sudan black B, waterborne polyurethane, polymeric dye, metal coatings

## Abstract

Colored waterborne polyurethanes have been widely used in paintings, leathers, textiles, and coatings. Here, a series of black waterborne polyurethanes (WPUs) with different ratios of black dye, Sudan Black B (SDB), were prepared by step-growth polymerization. WPU emulsions as obtained exhibit low particle sizes and remarkable storage stability at the same time. At different dye loadings, essential structural, statistical and thermal properties are characterized. FTIR (fourier transform infrared) spectra indicate that SDB is covalently linked into waterborne polyurethane chains. All of the WPUs with covalently linked SDB show better color fastness and resistance of thermal migration than those with SDB mixed physically. Besides, WPUs incorporated SDB covalently with different polymeric diols, polytetramethylene ether glycol (PTMG), polypropylene glycol (PPG), poly-1, 4-butylene adipate glycol (PBA) and polycaprolactone glycol (PCL), were prepared to obtain different properties to cater to a variety of practical demands. By a spraying method, the black WPUs can be directly used as metal coatings without complex dyeing process by simply mixing coating additive and other waterborne resins, which exhibit excellent coating performance.

## 1. Introduction

Recently, colored polymeric dyes, especially back dyes, have been widely used in textiles, ink, coatings, leathers and photoelectric materials [1,2,3,4,5,6]. Generally, the strategy on developing polymeric dyes is usually to physically mix micromolecular dyes with polymeric matrices by ionic bonds, hydrogen bonds or Van der Waals force. However, there is a thorny problem that, with time elapsing, the dyes may migrate and aggregate, leading to color fading of materials due to the noncovalent bond interaction between matrices and dyes. Moreover, plenty of black dyes containing benzidine groups have a potential risk to humans and environment [7,8,9]. Therefore, low-toxic and environmentally friendly materials are of great importance.

An effective method to solve the problem is to chemically link micromolecular dyes to polymeric main chains [10,11] or side chains [12] by various chemical reactions. Generally speaking, polymeric dyes are safe and nontoxic for humans because they cannot be absorbed by skin owing to their large molecular dimension, excellent chemical and thermal stability. Moreover, polymeric dyes with tunable molecular structures exhibit great compatibility and strong binding force with fibers. In the past decades, many researchers have been devoted to investigating polymeric dyes. For example, in the 1980s, Marechal et al. studied systemically on polymeric dyes for the first time [13,14,15]. Recently, many polymeric dyes have been prepared by incorporating chromophores into common polymeric materials, such as polyacrylates, polyethlene, polyamide and polymaleic acid, to enlarge the application fields [12,16,17,18,19]. Waterborne polyurethanes (WPUs) as a kind of highly versatile polymeric material with excellent environment-friendly and low-toxic properties have been widely used as coatings, leathers, adhesives, and paints [20,21,22]. By a facile polycondensation reaction, a lot of colored WPUs are developed by chemically incorporating micromolecular dyes into polyurethane matrices, which could show great migration resistance and color fastness while not obviously changing the intrinsic characteristic of the polymer materials [23,24,25,26]. However, there are few of reports on black dyes because of incomplete purity of black for purely organic dyes. 

Here, A series of novel black WPU dyes with different ratios of black dye, Sudan Black B (SDB), were prepared by polycondensation reaction, which exhibit good migration resistance, storage stability and heat resistance, while, at the same time, not causing significant aggregation/phase separation between micromolecular dyes and WPU matrices. Besides, Black WPUs with different polymeric diols are also investigated serving as black metal coatings, which exhibit excellent coating performance. 

## 2. Experimental

### 2.1. Materials 

Isophorone diisocyanate (IPDI) was purchased from Bayer Co., Ltd. (Leverkusen, Germany) Polytetramethylene ether glycol (PTMG, Mn = 2000) and polypropylene glycol (PPG, Mn = 2000) were supplied by Mitsubishi Co., Ltd. (Tokyo, Japan). Poly-1,4-butylene adipate glycol (PBA, Mn = 2000) was obtained from Qingdao Xinyutian Chemical Co., Ltd. (Qingdao, China). Polycaprolactone glycol (PCL, Mn = 2000) was supplied by Daicel Corporation (Kobe, Japan). All of the glycols were thoroughly dehydrated at 110 °C before use. 2, 2-dimethylolpropionic acid (DMPA) and Sudan black B (SDB, C.I.26150) were purchased form Aladdin Reagent Co., Ltd. (Shanghai, China). 1,4-Butanediol (BDO), Dibutyltin dilaurate (DBTDL), triethylamine (TEA), were purchased from Sinopharm Chemical Reagent Co., Ltd. (Hefei, China). BYK-025 and BYK-331 were acquired from BYK-Chemie GmbH Co., Ltd. (Wesel, Germany). ACRYSOL^TM^ RM-8W was provided by Rohm&Haas Co., Ltd. (Philadelphia, America). Other reagents were obtained from Energy Reagent Co., Ltd. (Shanghai, China), and used as received.

### 2.2. Methods

Fourier transform infrared (FTIR) spectra were recorded on a Bruker Tensor27 FTIR spectrometer (Bruker Co., Ltd., Karlsruhe, Germany) in the range of 4000–500 cm^−1^ using the thin WPU films (less than 20 μm in thickness) prepared by coating SDB-WPU emulsions on a potassium bromide (KBr) flake and then evaporating water by heating under an infrared lamp. 

Ultraviolet-visible (UV-vis) spectra of SDB and WPU dispersion were measured by UV-3600 spectrophotometer (Shimadzu Co., Ltd., Kyoto, Japan) in dimethylformamide (DMF) and water at 298 K. UV-vis spectra of WPU films were recorded on UV-vis-NIR spectrometer (Shimadzu Co., Ltd., Kyoto, Japan) ranging from 240 nm to 1200 nm at 298 K. 

Dynamic light scattering (DLS): The particle size distribution of WPU emulsion were carried out on a Zetasizer Nano ZS-90 (Malvern Co., Ltd., Worcestershire, the United Kongdom) by dynamic light scattering (DLS) at room temperature. 

Gel permeation chromatography (GPC) analyses were investigated by a Waters GPC instrument system (Waters Co., Ltd., Milford, Massachusetts, America) and calibrated with linear polystyrene, at a constant column temperature of 35 °C using tetrahydrofuran (THF) as eluent with a flow rate of 0.6 mL/min.

Differential scanning calorimetric (DSC) curves were recorded via a Mettler-Toledo DSC (Mettler-Toledo Co., Ltd., Zurich, Switzerland) at a constant heating rate of 10 °C/min from −65 °C –180 °C under N_2_ atmosphere. 

Thermogravimetric analysis (TGA) curve was collected by Shimadzu TGA-50 (Shimadzu Co., Ltd., Kyoto, Japan) under N_2_ atmosphere at a constant heating rate of 10 °C/min from 25 °C to 700 °C. 

WPU films were prepared by pouring WPU emulsions onto some polytetrafluoroethylene panels, and the panels were left at 25 °C for 48 h to obtain a series of homogeneous thin films with a thickness of 1 mm. The as obtained films were characterized without further annealing process.

**Scheme 1:**
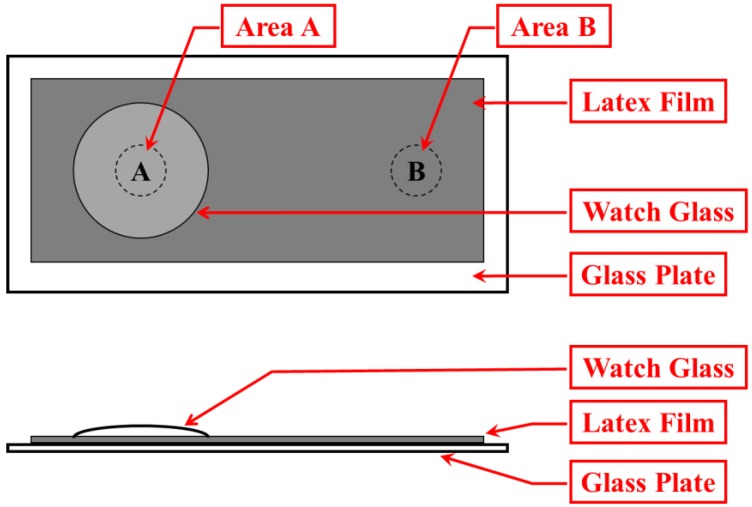
Evaluation model for thermal migration of the dye.

The migration ratio (Mp) was used to evaluate migration resistance of SDB-WPU. A glass plate was coated with SDB-WPU latex to form uniform film and divided into two areas (Area A and Area B), as shown in Scheme 1. Area A was clamped by a watch glass tightly, while Area B was exposed to the air. Then, the glass plate was kept at 60 °C for 24 h. The films in Area A and Area B were selected and dissolved into DMF at the same concentration and the absorbance was measured by UV-3600 spectrophotometer. Mp was calculated using the followed formula:

Mp = [(A_maxB_ − A_maxA_)/A_maxA_] × 100% (1)

where A_maxA_ and A_maxB_ are the maximum absorbance (A_max_) of the SDB-WPU in DMF in Area A and Area B, respectively.

Preparation of the black coatings: SDB-WPU, defoamer, thickener, leveling agent and deionized water were added into a dispersion machine. After stirring at high speed until a well-blended latex forms and then being left to stand for 30 min, the latex was sprayed on the surface of steel plate and kept at 100 °C for 20 min.

The physical properties of the SDB-WPU black coatings were evaluated according to standard test methods (impact strength GB/T 1732-93, Gloss GB/T 1743-89, adhesion force GB/T 1720-89, pencil hardness GB/T 6739-2006, and water resistance GB/T 23999-2009).

### 2.3. Synthesis of SDB-WPUs

**Scheme 2:**
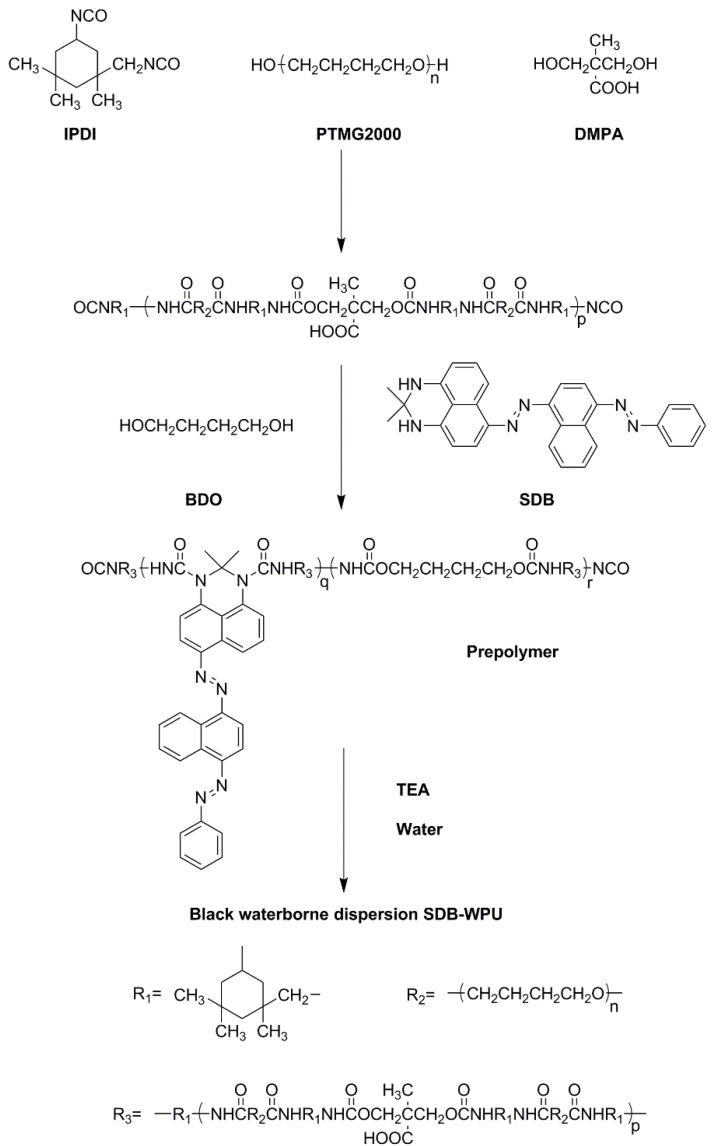
Synthetic processes of SDB-WPU (Sudan Black B-waterborne polyurethanes).

The preparation processes of SDB-WPUs are shown in Scheme 2. IPDI and PTMG (Mn = 2000) were added to a three-neck round-bottom flask (specific mass ratios are presented in Table 1). Then, the mixture was heated at 90 °C for 2 h under an N_2_ atmosphere (NCO content was determined using a standard di-*n*-butylamine titration test method [27]). Subsequently, the mixture was cooled down to 80 °C; a specified amount of DMPA was added; and it was again heated to 80 °C for approximately 2 h until the content of the NCO group in the mixture reached the expected theoretical value. The calculated amounts of BDO and SDB were added at 70 °C and allowed to react for 4 h with a trace amount of DBTDL (0.05–0.1 wt %) as a catalyst. A moderate amount of acetone was required at this stage to reduce the viscosity in the course of polymerization (the weight ratio of prepolymer and acetone is 7:3). TEA as a neutralization agent (neutralization ratio is 100%) was added at 40 °C to react with carboxyl group for 5 min to form a NCO-terminated WPU prepolymer (the molar ratio of –NCO and –OH is around 1–1.1). Finally, specific deionized water was poured into the mixture with the shearing rate of 3000 r/min to form WPU emulsion. A black aqueous dispersion was obtained after the acetone was removed from the WPU emulsion using a rotary evaporator in vacuum. The solid content of the obtained WPU emulsion was approximately 30 wt %. By this method, a series of SDB-WPUs were synthesized, as illustrated in Table 1.

## 3. Results and Discussion

### 3.1. UV-Vis Spectra of SDB

**Figure 1:**
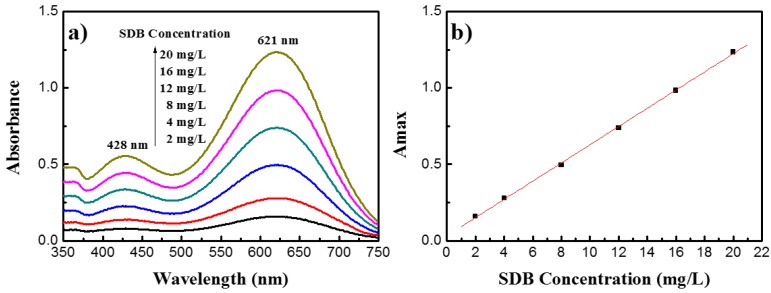
(**a**) UV-vis absorption spectra of SDB (Sudan Black B) with different dye concentrations in DMF (dimethylformamide). (**b**) The linear relationship of the Amax (621 nm) with different dye concentration.

Sudan black B (SDB) is a highly coloration-efficient and cost-effective dye, with high molar absorptivity. The UV-vis spectra of SDB with different concentrations are measured in *N*, *N*-dimethylformamide (DMF). As Figure 1a shows, SDB has an almost total absorption in the visible region with an intense absorbance around 621 nm and a minor absorbance centered at 428 nm, indicating that SDB solutions are nearly blue-black in dilute solution. Obviously, the maximum absorbance (A_max_) at 621 nm intensifies from 0.159 to 1.235 as the concentration of SDB in DMF increases from 2 mg/L to 20 mg/L (Figure 1b), which lines up with Beer-Lambert Law that can be used to calculate the SDB concentration in SDB-WPU.

### 3.2. Structural Analysis of SDB-WPUs 

**Figure 2:**
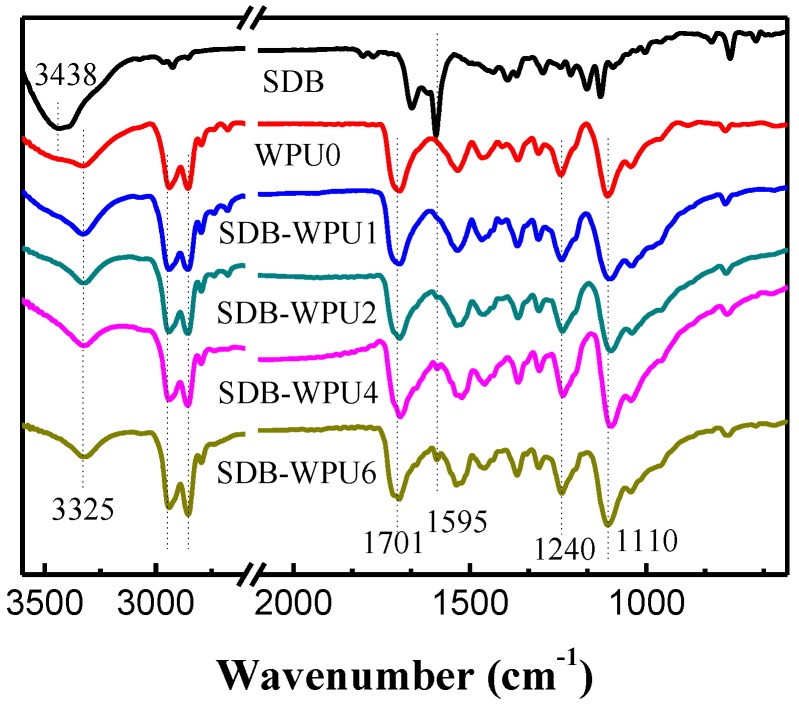
FTIR spectra of SDB and SDB-WPU with various dye contents on KBr plates.

SDB-WPUs were synthesized from the reaction between –NH/–OH and –NCO groups according to previously reported polycondensation reaction (Scheme 2) [20,28]. Briefly, there are two main steps in the course of polymerization. One is the reaction of diols and diisocyanates to obtain prepolymers containing SDB. The other is the emulsifying process of the prepolymers with a high shear speed after trimethylamine is added. Figure 2 shows the FTIR spectra of SDB-WPUs with different ratios of SDB (0–6%). Compared to WPU without SDB (WPU0), the characteristic absorption peak at 1595 cm^−1^ (N=N) appears and enhances with increasing SDB loadings. Meanwhile, the representative N–H vibration at 3430 cm^−1^ disappears in all of the WPU films, suggesting that SDB has been covalently linked to the WPU chains. Other characteristic absorption peak assignments for SDB-WPUs include: 3325 cm^−1^ (ν_NH_), 2860 cm^−1^ and 2940 cm^−1^ (ν_CH2_ and ν_CH3_), 1701 cm^−1^ (ν_C=O_), 1240 cm^−1^ (ν_C–O_ in carbamate group) and 1110 cm^−1^ (ν_C–O–C_ in PTMG), indicating that SDB-WPUs were synthesized successfully.

**Figure 3:**
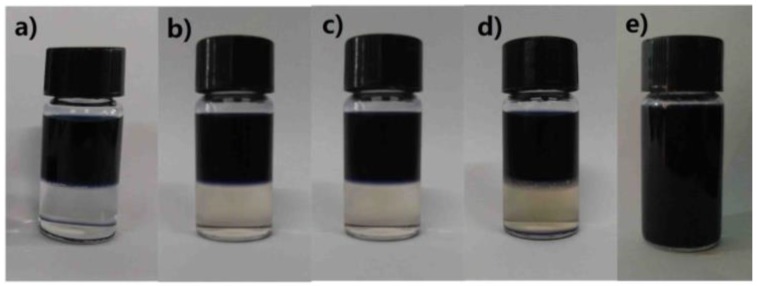
Graphs of: SDB-WPU2 (**a**–**d**); and SDB+WPU (physical mix of 2 wt % SDB and pure WPU) (**e**) migration between aqueous phase and CH_3_Cl phase at different moments (t_s_). (**a**) t_s_ = 0 h; (**b**) t_s_ = 6 h; (**c**) t_s_ = 12 h; (**d**) t_s_ = 24 h; and (**e**) t_s_ = 5 min.

To further demonstrate that SDB is chemically linked to polymeric main chains, the investigation on macroscopic migration of the SDB-WPU between water and solvent phases is conducted. The alteration of the color in two phases is recorded by digital camera at different moments, as illustrated in Figure 3. To water emulsion are added chloroform to illustrate whether SDB can be extracted. As Figure 3 shows, after stirring, chloroform does not immediately turn black. However, after 24 hours standing, there is no obvious change in chloroform with slight black, which are caused by few SDB-WPUs transferring from water to organic phase. As we all know, SDB as micromolecular dye has high mobility, which should migrate into water immediately after intense stirring because SDB is hydrophobic. Therefore, the slight change in chloroform ascribed to migration of SDB-WPU is reasonable, further indicating that SDB is covalently incorporated into WPUs. The mechanical stability in Table 2 also suggests that SDB is reacted into WPUs due to no obvious alteration in emulsion state, which means that no hydrophobic SDB aggregates precipitates after centrifugal sedimentation.

### 3.3. Statistical Properties of SDB-WPUs

The specific ratio of each component is shown in Table 1. The particle diameters measured by DLS of emulsions are shown in Table 2. All of the WPU dispersions exhibit tiny average particle size (< 50 nm). As the SDB ratio increases from 1% to 6%, the average particle size increases from 19.79 nm to 43.32 nm, ascribed to the enhanced hydrophobicity of SDB-WPU due to multi-aromatic rings in SDB. Besides, multi-aromatic structures and large size of SDB may prevent polymeric chains from entangling to small radius. To the best of our knowledge, smaller particle sizes show better emulsion stability. After more than six months, the dispersion remains homogeneous without any precipitates (Table 2). Moreover, there is no obvious change in emulsion state after 30 min centrifugation at 3000 r/min (Table 2), which demonstrates that SDB-WPUs are mechanically stable. 

The molecular weight information of SDB-WPUs is characterized by gel-permeation chromatography (GPC, Table 2). In Table 2, all of the SDB-WPUs show broad molecular weight distributions with polydispersity indices around 2–4. It is worth noting that molecular weight information of SDB-WPUs is not very reliable due to the presence of extremely polar carboxylate and ammonium groups. 

### 3.4. DSC and TG Analyses

**Figure 4:**
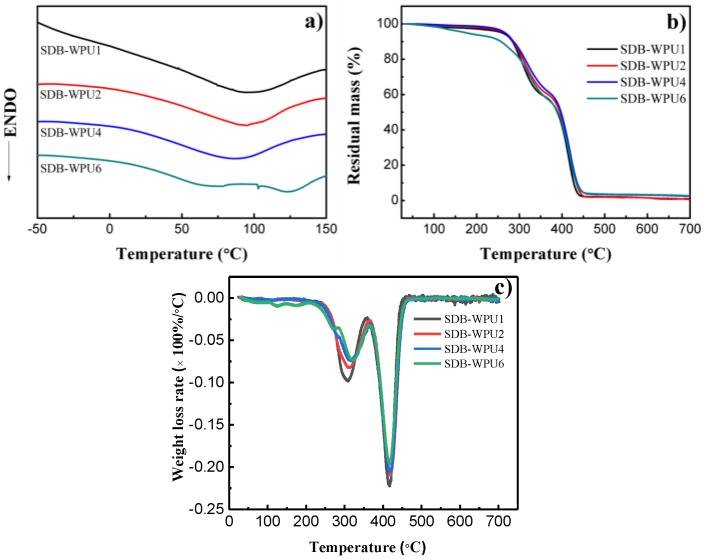
Thermal characterization of SDB-WPUs with various SDB contents via: DSC (**a**); TG (**b**); and DTGA (**c**) measurements.

Figure 4 shows the thermal properties of SBD-WPUs with different contents. From Differential Scanning Calorimetry (DSC) curves (Figure 4a) (all of the samples were measured without the elimination of the thermal history), a broad endothermic peak appears obviously in the range of 50–100 °C for all of the samples, which is not a typical glass transition, which usually shows a slope between two platforms [29]. The broad peak is due to the disappearance of short-range ordered structures in the hard segments [30]. There is a new endothermic peak around 125 °C for SDB-WPU6, which is due to the damage of microcrystalline structures organized in the process of film formation. Thermogravimetric (TG) analysis is shown in Figure 4b. All films show three thermal decomposition courses. The slow descending slopes from the 80 to 250 °C due to the disappearance of small molecules such as triethylamine and water. The steep slopes showing in the 250 °C–350 °C region are the breakage of allophanate and carbamate bonds. The last stage appears in the 350 °C–460 °C region where C–C bonds in the soft segment (PTMG) of WPUs decompose. It is noteworthy that SDB-WPU6 exhibits more clear decrease in the first decomposition stage. To make SDB-WPU6 (solid content is ~32%) disperse homogenously, more deionized water was added due to the incorporation of more rigid and hydrophobic SDB compared to other samples. Therefore, there may be more residual water in the film after being left at 25 °C for 48 h. With increasing SDB loadings, the maximum hard-segment decomposition temperature rises from 306 °C to 314 °C by a derivative thermogravimetric analysis (DTGA) (Figure 4c), which may be caused by more and more rigid phenyl rings in the hard segments, while the maximum soft-segment decomposition temperature remains at 418 °C, indicating that there is microphase separation to some extent between soft segments and hard segments.

### 3.5. UV-Vis Spectra Analysis of SDB-WPUs

**Figure 5:**
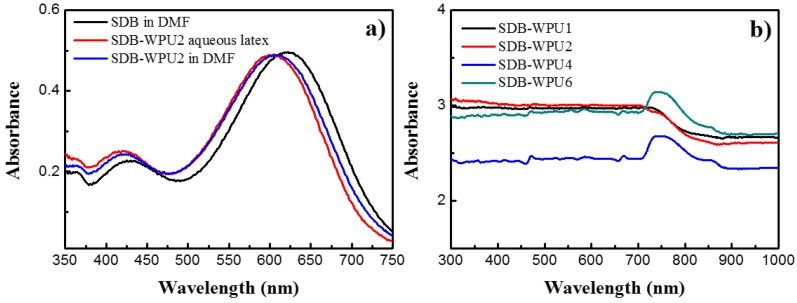
(**a**) UV-vis absorption spectra of SDB in DMF, SDB-WPU2 aqueous emulsion and SDB-WPU2 in DMF (blue) with the same concentration of SDB. (**b**) UV-vis absorption spectra of the SDB-WPU films dispersed in BaSO_4_.

Figure 5 shows the UV-vis spectra of SDB-WPUs. Compared to the UV-vis spectra of pure SDB in DMF solution, it is clearly found that, when SDB is introduced into the WPU chains, the maximum absorption of SDB shifts from 623 nm to 603 nm, which likely results from the influence of conjugate effect between aromatic amino in SDB and allophanyl in WPUs, which weakens electron-donating ability of aromatic amino in SDB. It can be illustrated by dissolving SDB-WPU films in DMF solution, in which maximum absorption wavelength is almost consistent with the SDB-WPU aqueous emulsion, excluding the possible impact induced by the polarity of solvent. Figure 5b shows the UV-vis spectra of SDB-WPU films. All of the SDB-WPU films exhibit complete, intense absorption in the visible region in comparison to solution state, which is almost pure black, as observed by the naked eye. 

### 3.6. Analyses of SDB-WPUs with Different Polymeric Diols

To investigate the influence of soft segments on physical properties of black WPUs, different polymeric diols (PTMG, PBA, PPG, PCL) are used to prepare WPUs with constant SDB content (2%, wt/wt). All of the WPU emulsions show the particle diameters around 10–20 nm, suggesting the excellent dispersion, storage and mechanical stability (Table 2). All of the samples exhibit no clear change after centrifuging for 30 min at 3000 r/min or sitting for six months. 

**Figure 6:**
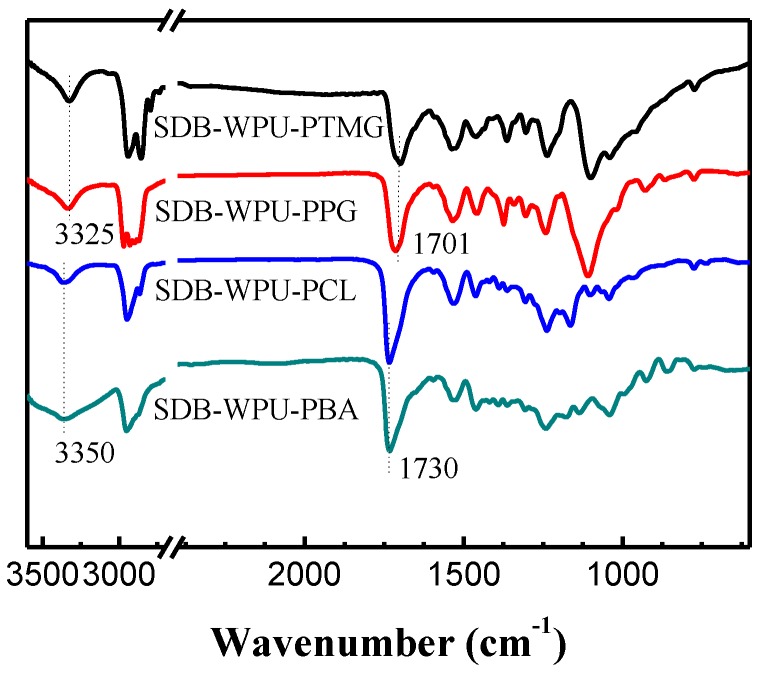
FT-IR spectra of SDB-WPUs with various polymeric diols on KBr plates.

The FT-IR spectra of WPUs with different polymeric diols are presented in Figure 6. Consistent with the description above, SDB is covalently attached into WPU chains due to the disappearance of N–H stretching vibration of SDB and the appearance of the representative N–H vibration at 3325 cm^−1^ in polyether-based WPUs (SDB-WPU-PTMG and SDB-WPU-PPG) and at 3350 cm^−1^ in polyester-based WPUs (SDB-WPU-PCL and SDB-WPU-PBA). The differences between polyether-based WPUs and polyester-based WPUs may be ascribed to their different microenvironments, such as the different degree of microphase separation and amounts of hydrogen bonds. A similar phenomenon is presented in stretching vibration of C=O (1730 cm^−1^ in polyester-based WPUs and 1701 cm^−1^ in polyether WPUs). Other characteristic absorption peak assignments for SDB-WPUs with different polymeric diols are similar to Figure 2: 2860 cm^−1^ and 2940 cm^−1^ (ν_CH2_ and ν_CH3_), 1240 cm^−1^ (ν_C–O_ in carbamate group) and 1110 cm^−1^ (ν_C–O–C_ in soft segments), suggesting that SDB-WPUs were synthesized successfully.

**Figure 7:**
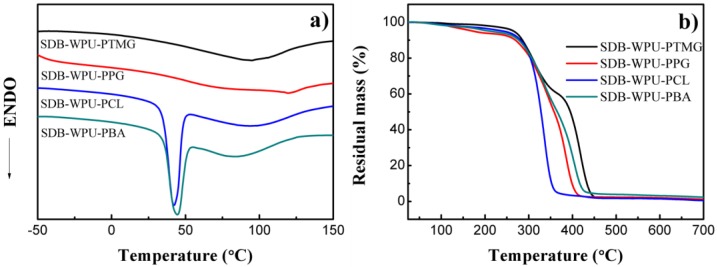
Thermal characterization of SDB-WPUs with various polymeric diols via: DSC (**a**); and TG (**b**) measurements.

The thermal properties of SDB-WPUs with various polymeric diols are characterized by DSC and TG curves (Figure 7). Similar results line up with the discussion above. The difference in DSC (Figure 7a) is that a typical endothermic peak appears at 40–50 °C in SDB-WPU-PCL and SDB-WPU-PBA due to the melting of crystalline structure of the polyester in PCL and PBA. There is a clear difference in third decomposition stage caused by different thermal resistance of polymeric diols and degree of microphase separation (Figure 7b) [31,32,33]. According to the TG curves, SDB-WPU-PTMG, with the highest starting and soft-segment decomposition temperatures, shows the best thermal resistance, while SDB-WPU-PBA exhibits the poorest thermal resistance. The remaining two kinds of WPUs have similar thermal resistances.

**Scheme 3:**
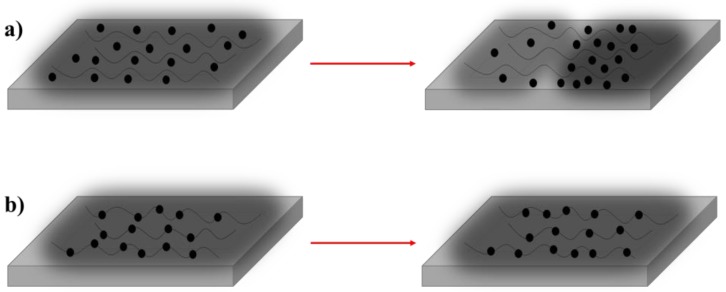
Thermal migration models of: dye-doped (not covalently bonded) WPUs (WPU+SDB) (**a**); and dye-linked WPUs (SDB-WPUs) (**b**).

As we all know, dyes are physically mixed into polymeric coatings influenced by dispersants in that dyes tend to migrate from the coatings interior to aggregate on the coatings surface due to weak binding force. To evaluate the migration property of SDB-WPUs, the same experiments were carried out with the sample formed by physically mixing WPU with SDB (WPU+SDB) as a control. As provided in Table 3, Mp values of SDB-WPUs and WPU+SDB are 7%–8% and 31.0%, respectively, manifesting that SDB-WPUs exhibit greater thermal-migration resistance than WPU+SDB. Shown in Scheme 3 are the models of thermal migration of dye-doped WPU and dye-incorporated WPU. SDB tends to migrate and aggregate to a certain area due to enhanced free volumes and weak binding force (Scheme 3a), which may cause inhomogeneous color distribution. Migration process occurs in both the interior and surface of SDB-WPU films. For example, SDB in the interior may migrate to surface and then aggregate to an area in the surface leading to the inhomogeneity of black. However, the mobility of SDB is confined due to the covalent binding of –NH in SDB and –NCO, resulting in homogeneous dispersity of SDB in WPU. The black SDB-WPU obtained by covalent link with excellent migration-resistance property can be applied effectively in polyurethane coatings, while traditional preparation method via mixing polyurethane resin with micromolecular dyes cannot easily achieve such remarkable migration resistance.

### 3.7. Application in Metal Coatings

**Figure 8:**
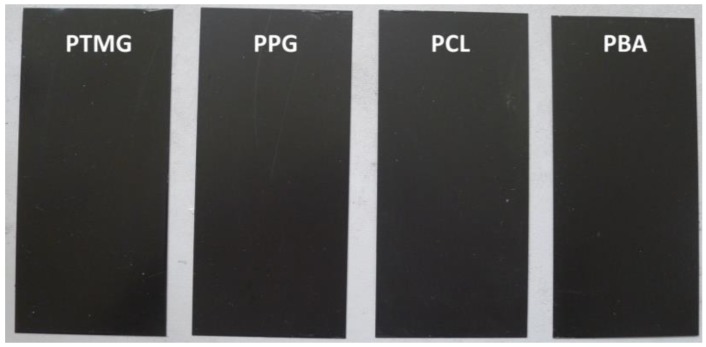
Black SDB-WPU coatings on the surface of steel plate with different soft segments: PTMG (polytetramethylene ether glycol); PPG (polypropylene glycol); PCL (polycaprolactone glycol); and PBA (poly-1, 4-butylene adipate glycol).

Black SDB-WPU coatings can easily be prepared by spraying the mixture on the metal surface after compounded with other waterborne resins and coatings additives (Table 4). The coating properties of the SDB-WPUs are illustrated in Table 5. All of the film coatings show smooth and bubble-free surfaces with glossiness around 80–90. Because of the chemically linking reaction, SDB is uniformly distributed into the polyurethanes without dyes aggregating and phase separation (Figure 8), resulting in excellent coatings performance. To further investigate the properties of SDB-WPU coatings, adhesion force, pencil hardness, impact strength and water resistance are tested. Slight difference can be observed in these properties, attributed to the change of polymeric diols, which are able to significantly influence degree of microphase separation, flexibility, rigidity and crystallinity. Consequently, different black WPU coatings with SDB linked chemically may be developed by changing the kind of polymeric diol to cater to a specific applied situation.

## 4. Conclusions

In summary, a series of black WPUs from Sudan black B (SDB) were prepared. The obtained SDB-WPU emulsions show remarkable storage and mechanical stability. Compared to the traditional method by physically mixing dyes with polymeric substrates, SDB-WPUs also exhibit excellent thermal-migration resistance and color fastness, in that SDB is chemically linked to WPU chains. Besides, incorporation of SDB does not obviously change the thermal properties of WPUs. By altering different polymeric diols, all of the SDB-WPUs show some differences in glossiness and adhesion force but maintain their excellent storage and mechanical stability, and great thermal resistance. Therefore, different black WPU coatings with SDB linked chemically may be developed by changing the kind of polymeric diol to cater to a specific applied situation.

## Figures and Tables

**Table 1:** The specific ratio of each component in SDB-WPUs (Sudan Black B-waterborne polyurethanes).

Sample	Polymeric Diol	Polymeric Diol /g	IPDI /g	DMPA /g	SDB /g	BDO /g	SDB (wt %)
SDB-WPU1	PTMG	25	12	2.3	0.42	1.8	1%
SDB-WPU2	PTMG	25	12	2.3	0.83	1.7	2%
SDB-WPU4	PTMG	25	12	2.3	1.7	1.55	4%
SDB-WPU6	PTMG	25	12	2.3	2.6	1.4	6%
SDB-WPU-PTMG	PTMG	25	12	2.3	0.83	1.7	2%
SDB-WPU-PPG	PPG	25	12	2.3	0.83	1.7	2%
SDB-WPU-PCL	PCL	25	12	2.3	0.83	1.7	2%
SDB-WPU-PBA	PBA	25	12	2.3	0.83	1.7	2%

**Table 2:** The various statistical properties of SDB-WPUs.

Sample	Average Particle Size	Storage Stability	Mechanical Stability	Mn ^a^	PDI ^b^
SDB-WPU1	19.79 nm	Unchanged	Unchanged	16,100	3.61
SDB-WPU2	21.38 nm	Unchanged	Unchanged	16,300	3.70
SDB-WPU4	41.69 nm	Unchanged	Unchanged	14,400	3.63
SDB-WPU6	43.32 nm	Unchanged	Unchanged	13,200	3.17
SDB-WPU-PTMG	21.38 nm	Unchanged	Unchanged	16,300	3.70
SDB-WPU-PPG	19.21 nm	Unchanged	Unchanged	16,000	2.79
SDB-WPU-PCL	13.40 nm	Unchanged	Unchanged	18,900	2.85
SDB-WPU-PBA	13.97 nm	Unchanged	Unchanged	17,400	2.67

**^a^** Number, average molecular weights of SDB-WPUs measured in THF; ^b^ Polydispersity index (*M*_w_/*M*_n_).

**Table 3:** Thermal-migration property of SDB-WPUs and WPU+SDB.

Samples	A_1_^a^	A_2_^b^	M_p_ (%) ^c^
WPU+SDB	0.332	0.435	31.0%
SDB-WPU-PTMG	0.309	0.332	7.4%
SDB-WPU-PPG	0.276	0.298	8.0%
SDB-WPU-PCL	0.327	0.351	7.3%
SDB-WPU-PBA	0.252	0.271	7.5%

**^a^** maximum absorbance of SDB-WPUs in area A; ^b^ maximum absorbance of SDB-WPUs in area B; ^c^ migration ratio of SDB-WPUS.

**Table 4:** Formulation of black SDB-WPU coatings.

Entry	Function	wt %
SDB-WPU	Resin	95
BYK-025	Defoamer	0.5
BYK-331	Leveling additive	0.3
ACRYSOLTM RM-8W	Thickener	1.2
Deionized water	-	3
Total	-	100

**Table 5:** The properties of black waterborne polyurethane coatings with various polymer diols.

Property	SDB-WPU-PTMG	SDB-WPU-PPG	SDB-WPU-PCL	SDB-WPU-PBA
Appearance	Smooth, No bubbles	Smooth, No bubbles	Smooth, No bubbles	Smooth, No bubbles
Gloss (60°)	83	83	86	87
Adhesion force (grade)	0	1	0	0
Pencil hardness	2B	3B	B	2B
Impact strength (kg/cm)	50	50	50	50
Water resistance (24 h)	Unchanged	Unchanged	Unchanged	Unchanged
